# Molecular and Source-Specific Profiling of Hospital *Staphylococcus aureus* Reveal Dominance of Skin Infection and Age-Specific Selections in Pediatrics and Geriatrics

**DOI:** 10.3390/microorganisms11010149

**Published:** 2023-01-06

**Authors:** Kamaleldin B. Said, Naif Saad Alghasab, Mohammed S. M. Alharbi, Ahmed Alsolami, Mohd Saleem, Sulaf A. Alhallabi, Shahad F. Alafnan, Azharuddin Sajid Syed Khaja, Taha E. Taha, on behalf of the Ha’il COM Research Unit Group

**Affiliations:** 1 Department of Pathology, College of Medicine, University of Ha’il, Ha’il 55476, Saudi Arabia; 2 Genomics, Bioinformatics and Systems Biology, Carleton University, 1125 Colonel-By Drive, Ottawa, ON K1S 5B6, Canada; 3 Department of Cardiology, College of Medicine, University of Ha’il, Ha’il 55476, Saudi Arabia; 4 Department of Internal Medicine, College of Medicine, University of Ha’il, Ha’il 55476, Saudi Arabia; 5 Department of Epidemiology, John Hopkins Bloomberg School of Public Health, Baltimore, MD 21205, USA

**Keywords:** clinical *S. aureus*, skin carriage, geriatric-MRSA pneumonia, endogenous-*S. aureus*, MSSA and MRSA lineages

## Abstract

*Staphylococcus aureus* is a major human-associated pathogen that causes a wide range of clinical infections. However, the increased human dynamics and the changing epidemiology of the species have made it imperative to understand the population structure of local ecotypes, their transmission dynamics, and the emergence of new strains. Since the previous methicillin-resistant *S. aureus* (MRSA) pandemic, there has been a steady increase in global healthcare-associated infections involving cutaneous and soft tissue and resulting in high morbidities and mortalities. Limited data and paucity of high-quality evidence exist for many key clinical questions about the pattern of *S. aureus* infections. Using clinical, molecular, and epidemiological characterizations of isolates, hospital data on age and infection sites, as well as antibiograms, we have investigated profiles of circulating *S. aureus* types and infection patterns. We showed that age-specific profiling in both intensive care unit (ICU) and non-ICU revealed highest infection rates (94.7%) in senior-patients > 50 years; most of which were MRSA (81.99%). However, specific distributions of geriatric MRSA and MSSA rates were 46.5% and 4.6% in ICU and 35.48% and 8.065% in non-ICU, respectively. Intriguingly, the age groups 0–20 years showed uniquely similar MRSA patterns in ICU and non-ICU patients (13.9% and 9.7%, respectively) and MSSA in ICU (11.6%). The similar frequencies of both lineages in youth at both settings is consistent with their increased socializations and gathering strongly implying carriage and potential evolutionary replacement of MSSA by MRSA. However, in age groups 20–50 years, MRSA was two-fold higher in non-ICU (35%) than ICU (18.6%). Interestingly, a highly significant association was found between infection-site and age-groups (*p*-value 0.000). Skin infections remained higher in all ages; pediatrics 32.14%, adults 56%, and seniors 25% while respiratory infections were lower in pediatrics (14.3%) and adults (17%) while it was highest in seniors (38%). Blood and “other” sites in pediatrics were recorded (28.6%; 25%, respectively), and were slightly lower in adults (18.6%; 8.6%) and seniors (14%; 22.8%), respectively. Furthermore, a significant association existed between infection-site and MRSA (Chi-Square Test, *p*-value 0.002). Thus, the common cutaneous infections across all age-groups imply that skin is a significant reservoir for endogenous infections; particularly, for geriatrics MRSA. These findings have important clinical implications and in understanding *S. aureus* profiles and transmission dynamics across different age groups that is necessary for strategic planning in patient management and infection control.

## 1. Introduction

*Staphylococcus aureus* is an important nosocomial pathogen. By the virtue of the species ability to rapidly adapt to humans, it continuously evolves and emerges into highly virulent lineages. It has been well established that superantigens of this species elicit cytokine storms leading to sever necrotizing pneumonia, necrotizing fasciitis, and invasive skin infections even in young and otherwise healthy people. The frequently changing transmission dynamics and clinical manifestations of *S. aureus* are similar to those of the SARS-CoV-2 and the monkeypox [1]. In extreme cases, *S. aureus* necrotizing pneumonia and disseminated intravascular coagulation can induce infectious Acute Respiratory Distress Syndrome (ARDS) shock leading to multi-organ failure [2]. Interestingly, while MRSA has been found to cause gastroenteritis and unilobar infiltrates, methicillin sensitive *S. aureus* (MSSA) was involved in airway hemorrhage, multilobar infiltrates, and ARDS [3]. Thus, knowledge of profiles of MRSA and invasive MSSA lineages in the region is important; particularly since they may aggravate the current pandemic viral infections. This study aims to determine the distributions of MRSA and MSSA infections and disease profiles in Ha’il hospitals, Saudi Arabia.

The changing epidemiology of *S. aureus* lineages have created several gaps in our understanding of this species’ subtle mechanisms of infection and transmission. During the last decade, pandemic emergence of *pvl* positive community acquired (CA)-MRSA, MRSA, and invasive MSSA lineages have paralysed global healthcare systems with mortality rates similar to that of AIDS, tuberculosis, and viral hepatitis combined [4,5,6,7,8,9]. *Staphylococcus aureus* has been evolving and emerging through decades, not in spite of, but surprisingly as a consequence of the advances in medical progress [10]. History explains a continuous evolutionary pattern of the original pandemic strains. A single major lineage that evolved into sub-lineages caused more invasive diseases than the combined rates of those caused by bacterial species with transformable genomes; nevertheless, estimates of *S. aureus* mortality rates were projected to exceed that of the HIV [6,11]. Despite the past bitter lesson, many cases are still considered indolent, consistent with the notion “those who do not remember the past are doomed to repeat it” as quoted by Chalmer et al., [12]. Although global MRSA pandemic outbreaks have declined, high morbidities and mortalities are still being reported globally due to sporadic outbreaks [13]. A comprehensive review of 15 clinical investigations showed that up to 74% of worldwide *S. aureus* infections were caused by different MRSA lineages in Europe [14], and another pandemic with an annual economic burden of $3.3 billion in the USA alone, was proposed. Thus, studies for more insights into *S. aureus* mechanisms of infection, transmission, acute nosocomial resistances, and carriage have become imperative in the context of the emerging viral pandemics with similar syndromes. Specifically, profiles of resistant strains circulating in hospitals and communities, antimicrobial resistance patterns, and carriage rates are not well defined in the region.

The declaration of COVID-19 as the new respiratory pandemic in early March 2020 introduced significant changes in the management of infections and co-infections [15]. Microbial co-infections during SARS-CoV-2 and monkeypox aggravate diseases, increase mortalities, and morbidities [16]. Coinfections significantly alter the pathophysiology of the disease and the patient recovery outcome [17,18]. Bacterial coinfections are considered more important than others based on experience with previous viral pandemics [19] where high mortalities in critically ill patients were reported [20]. However, there is a significant paucity in high quality data on established *S. aureus* infection patterns, strain profiles, and clinical characteristics. This makes it difficult to precisely estimate the role of the species in co-infection during COVID-19 and/or monkeypox. Of particular importance are the respiratory and skin lineages such as HA-MRSA and CA-MRSA. Susceptibility to infections by one of the MRSA lineages is ideally measured by their colonization of the nares and skin surfaces where their absence is a known negative indicator of the disease. It has been recently highlighted that MRSA nasal screening in non-COVID-19 patients is a useful antimicrobial stewardship to avoid unnecessary empiric MRSA therapy such as vancomycin [21,22]. Current guidelines for the treatment of pneumonia as per the American Thoracic Society (ATS) and Infectious Diseases Society of America (IDSA) recommended empiric MRSA coverage in patients at-risk [23,24]. While initiating appropriate empiric antibiotics in a timely manner is critical, identifying nasal and skin carriage status is equally important for specific initiation and de-escalation timings of anti-MRSA coverage therapy.

Community-acquired pneumonia (CAP) is most severe in communities and/or extended home-cares worldwide due to the septic necrotizing pneumonia [12,25,26]. *Staphylococcus aureus* hospital pulmonary sepsis has been postulated to correlate with ARDS for years until a recent study confirmed the direct involvement of MRSA and the role of FTY720 S-phosphonate in endothelial cell protection [27]. An estimated 30 million cases of lung sepsis annually have led to more than eight million deaths, i.e., 15–30% in high-income countries and 50% or higher in low-and middle-income countries [28]. It becomes serious when *pvl*-positive *S. aureus* (CA-MRSA lineage) are involved in infectious ARDS conditions. More importantly, clinical management is particularly challenging when the etiologic scenarios in ARDS and superantigenic CA-MRSA pneumonias are further complicated by similar respiratory COVID-19 and/or skin monkeypox syndromes. In recent years, with the increase in global population dynamics, a significant increase in community associated lung infections have occurred globally. Despite the remarkable progress made in advancing healthcare systems, pneumonia associated with lung sepsis remains burdensome in global public health [29,30]. Furthermore, the high complexity and costs associated with lung care complicates cases leading to high morbidity and mortality. Particularly, the clinical and economic burden of CAP is staggering, far-reaching, and expected to increase as new antibiotic resistance mechanisms emerge while the world’s population ages [31]. Therefore, a leading cause of death worldwide is sepsis, especially when developed as a dysregulated immune response to infectious pneumonia [32,33]. The potential risk of *S. aureus* in these cases is quite high.

Evolution of virulent strains and re-emergence of lineages associated to high morbidities and mortalities are being reported [13]. For instance, up to 74% of global *S. aureus* infections are caused by evolving new lineages [14]. Particularly, the evolution of invasive MSSA strains causing bloodstream infections is being increasingly reported [34]. Emergence of a single MSSA clone in Greece carrying high level resistances primarily to mupirocin (99%) has caused significant staphylococcal scaled skin syndromes in a setting; a total of 85% of the cases were impetigo [35]. Furthermore, the recent emergence of a new and the previously unreported clonal complexes of methicillin-resistant MRSA strains in the region are of concern [36,37,38,39].

It Is still not clear how *S. aureus* switches from a commensal to a life threatening pathogen and from human to animal associated lineages or vice versa despite significant evidence of a clonal core genome from intrinsic and highly polymorphic accessory genes [40,41,42,43]. The common genomic background of the species opens a new aspect in *S. aureus* epidemiology, i.e., understanding the principles underlying animal to human-to-human transmission dynamics and/or vice versa across different gender and age groups is key in its host- and tissue-specific adaptive emergences. For these reasons, regular hospital surveillance of *S. aureus* infections for local strain profiles, sources of transmissions across different age groups, and antimicrobial resistance is important. We have previously used surveillance programs to understand the rates of *S. aureus* infections followed by molecular differentiations of MRSA and MSSA lineages to identify dominant clonal lines in North America and the Middle East [44,45,46].

Unfortunately, significant variations occur in the rates of *S. aureus* surveillance programs in Middle Eastern and African countries (MENA) that make it difficult to develop an effective MRSA management program. A recent comprehensive report from January 2005 to December 2019 revealed great heterogeneity in MRSA rates. For instance, nasal MRSA colonization ranged from 2–16% in the Gulf Cooperation Council (GCC), 1–9% in the Levant, and 0.2–9% in North African Arab states. Clinical isolates of MRSA ranged from 9–38% in GCC, 28–67% in the Levant, and 28–57% in North African states. Studies demonstrated a wide clonal diversity in the MENA. In addition, significant diversities in clonal complexes and antimicrobial resistances were also seen in the region with variation in patterns depending on location and clonal type [47]. Thus, comprehensive, and accurate prevalence of *S. aureus* in the region is required in each country first, before vertical genotyping of dominant clones for local strain profiling. This study was intended to determine the distributions of hospital *S. aureus* and sources and infection patterns in different age groups, disease profiles, antibiogram patterns, and types of circulating MRSA and MSSA lineages across different patient groups in hospital units. In this study, we report on significant insight into the patterns of distributions of *S. aureus* hospital ecotype-lineages, i.e., the high prevalence of MRSA in the two extremes of life, i.e., the pediatrics and senior patients, similar coexistence of MRSA and MSSA in younger groups < 20 years at ICU, and the two-fold increase in MRSA at non-ICU patients >20 years old. In addition, we found a significant association to infection sites; particularly, the dominance of skin infections among patient groups.

## 2. Materials and Methods

### 2.1. Bacteriological Analysis, Patients’ Demographics, and Antimicrobial Susceptibility Testing

In this work, we analyzed all positive specimens for non-duplicate isolates of methicillin-resistant *S. aureus* (MRSA) and methicillin sensitive *S. aureus* (MSSA), obtained from clinical infections recovered from hospitals in Ha’il in the first quarter year of 2021. All data were collected from microbiology laboratory records, hospital medical records, and various sources within hospitals. The data included but was not limited to antibiotic sensitivity data, specimen types and collection sites, intensive care unit (ICU), and non-ICU ward, and age differences from King Khalid hospital.

### 2.2. Microbiological Analysis

In general, specimens were analyzed by routine bacteriology and standard antimicrobial sensitivity testing. Briefly, they were cultured to confirm primary identifications, preparations of inoculums for storage, and for automated testing. Isolates were identified by standard bacteriological methods and ID and susceptibility testing using automated systems. For non-automated procedures, specimens were aseptically collected in suitable transport media and swabs to the lab, processed immediately, and cultured using standard conditions and media under 37 °C incubations for 18 h. Bacterial isolates were kept in broth cultures at −80 °C for future reference and vertical studies. However, most of the work performed on automated systems mostly included BD Phoenix system (BD Biosciences, Franklin Lakes, NJ, USA) and MicroScan plus (Beckman Coulter, Brea, CA, USA) for the identification and antimicrobial sensitivity. Susceptibility was confirmed by culture and agar diffusions experiments as necessary. Susceptibility testing and breakpoint interpretive standards were carried out in accordance to the recommendations of Clinical and Laboratory Standard Institute (CLSI document M100S-26) [48].

### 2.3. Classifications as Multi-, Extremely- and Pan-Drug Resistant Bacteria (MDR, XDR, and PDR)

The standard definitions for acquired resistances classifications categorize hospital acquired MRSA isolates as multi drug-resistant (MDR) by the virtue of their methicillin resistance and resistance to beta lactams. However, this classification (i.e., MDR) does not apply to the community-acquired lineages (CA-MRSA) since they are known to be susceptible to beta lactams. Furthermore, extensive drug-resistant (XDR), and pan drug-resistant (PDR) are usually applied based on recommendations of European Centre for Disease Control. The MDR criteria for acquired resistance states non-susceptibility to at least one agent in three or more antimicrobial categories, XDR was defined as non-susceptibility to at least one agent in all but two or fewer antimicrobial categories (i.e., bacterial isolates remain susceptible to only one or two categories) and PDR was defined as non-susceptibility to all agents in all antimicrobial categories as reported by Magiorakos et al., [49]. Intrinsic resistances to particular drugs were not included. Criteria for defining *S. aureus* MDR classifications include one or more of the following to apply: 1. hospital acquired MRSA is always considered MDR by virtue of being an MRSA. 2. Non-susceptible to ≥1 agent in >3 antimicrobial categories.

### 2.4. Direct Multi-Gene Molecular Detection of S. aureus Lineages by GeneXpert System

The reason for using the advanced molecular system, the GeneXpert RT-PCR, is to address the problems of mutations during laboratory serial subculturing, long term in vitro passages, and to minimize the highly alert adaptive mutations and expressions of *S. aureus* genome in response to laboratory media and processes. We and others have established that these in vitro processes significantly alter the genetic profiles of the original genotype isolated from patients. Substantial experimental errors can be introduced when these isolates are used in downstream studies. Therefore, direct molecular detection from patient specimen allows for correct strain profile consistent with clinical characteristics, patient demographics, and disease categories recorded.

Direct molecular detection and characterizations were carried out in the latest versions of the Cepheid GeneXpert^®^ Dx system using the SA Complete and MRSA assay kits) using manufacturers recommendations and names and codes included in each kit. This system is equipped with mutli-gene molecular primers and reagent kits for robust automated direct detection, characterization, and differentiation of *S. aureus* lineages directly from specimens. The system uses built-in primers for *nuc spa*, *mecA* and the *mec* (SCC*mec*) gene direct detections from specimens. This test utilizes automated real-time polymerase chain reaction (PCR). Culturing was also done for further susceptibility testing as explained earlier. The GeneXpert Dx is all-in-one system that integrates sample purification, nucleic acid amplification, and detection of the target sequence in simple or complex samples using real-time PCR. It consists of an instrument, personal computer, and preloaded software for running tests and viewing the results. A single-use disposable self-contained cartridge with PCR reagents is inserted and inoculated directly with swabs/samples. In addition to avoiding environmental cues that alter the genome, cross-contamination between samples or during specimen collection or processing as well as cross-sequence contaminations in molecular tests are all remote since the cartridge is a disposable, closed, and self-contained kit. A sample processing control (SPC) and a Probe Check Control (PCC) are also included. The SPC is present to control for adequate processing of the target bacteria and to monitor the presence of inhibitor(s) in the PCR reaction. The PCC verifies reagent rehydration, PCR tube filling in the cartridge, probe integrity, and dye stability.

### 2.5. Statistical Analysis

Collected data were analyzed using Statistical Package for Social Sciences software (IBM SPSS; Version 24 SPSS version 23.0 for Windows (SPSS, Inc., Chicago, IL, USA). Descriptive and stratified analyses were conducted; we present absolute numbers, proportions, and graphical distributions. We conducted exact statistical tests for proportions and show *p*-values (based on Chi square test values) where appropriate (a *p*-value < 0.05 was considered statistically significant).

## 3. Results

**Figure 1:**
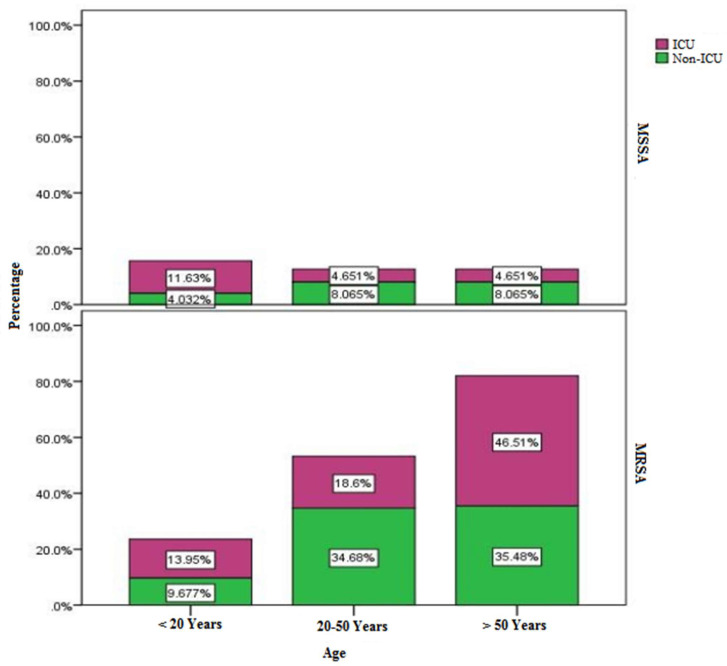
Age-group specific distributions of MRSA and MSSA isolates recovered from clinical specimens in ICUs, and other non-ICUs (inpatient and outpatient) settings in Ha’il hospitals, Saudi Arabia. Abbreviations: MRSA—Methicillin-resistant *Staphylococcus aureus*; MSSA– Methicillin-sensitive *Staphylococcus aureus*; ICU-intensive care unit; non-ICU-non-intensive care unit.

In this study, we have collected 195 isolates of *S. aureus* for clinical disease profiling, antibiogram patterns, and rates, molecular types of circulating MRSA and MSSA lineages in different hospital settings. Of these isolates, overall, 41% (*n* = 80) of the isolates were MSSA and the rest 60% (*n* = 115) were MRSA. However, 167 isolates were used for comparative examination of ICU and non-ICU infections across different age groups. As shown in Figure 1, the overall MSSA infection rates were lower among different age groups of patients in ICU and non-ICU settings compared to that of MRSA. Different age groups of patients revealed different patterns of *S. aureus* lineages and disease characteristics with lowest infection rates reported in pediatric patients.

### 3.1. Age-Specific Frequencies of S. aureus Lineages in ICU and Non-ICU

Figure 1 and Table 1 showed that in the first group (0 to 20 years old), the overall total MRSA isolation rate in both ICU and non-ICU settings was 23.6% (*n* = 39 of 167) while that of MSSA was 16% (*n* = 26 of 167). However, among this age group under ICU, 14% were positive for any type of MRSA infections while 11.6% had infections with invasive MSSA lineage. On the other hand, among the same aforementioned age group under other clinical settings than ICU (inpatient and outpatient), MRSA isolation rate of 9.7% was over two times higher than that of MSSA (4%). However, in the subsequent higher age groups, rates of *S. aureus* infections substantially increased as indicated by the results obtained below. In the second age group among the young and mid-aged patients (20–50 years old), the overall MRSA and MSSA rates in all hospital settings were 53.3% and 12.7%, respectively. Patients in the above age group under ICU units reported 18.6% and 4.6% of MRSA and MSSA infections, respectively. However, the overall rate of infections by the two lineages under non-ICU “other clinical settings than ICU” was 42.7% of which 34.68% of patients were positive for MRSA infections and 8.065% of them had MSSA infections. The third age group included patients over 50 years who had the highest frequency of overall *S. aureus* infections (94.7%). Among these, the overall total rates of the two lineages, MRSA and MSSA, in all settings were 81.99% and 12.7%, respectively. However, in ICU, 46.5% were MRSA and 4.6% were MSSA. On the other hand, under other non-ICU clinical settings, the senior patient group showed isolation rates of 35.5% and 8% MRSA and MSSA, respectively (Figure 1, Table 1).

### 3.2. Organ-Specific Distribution of Clinical S. aureus Infections among Different Age Groups

**Figure 2:**
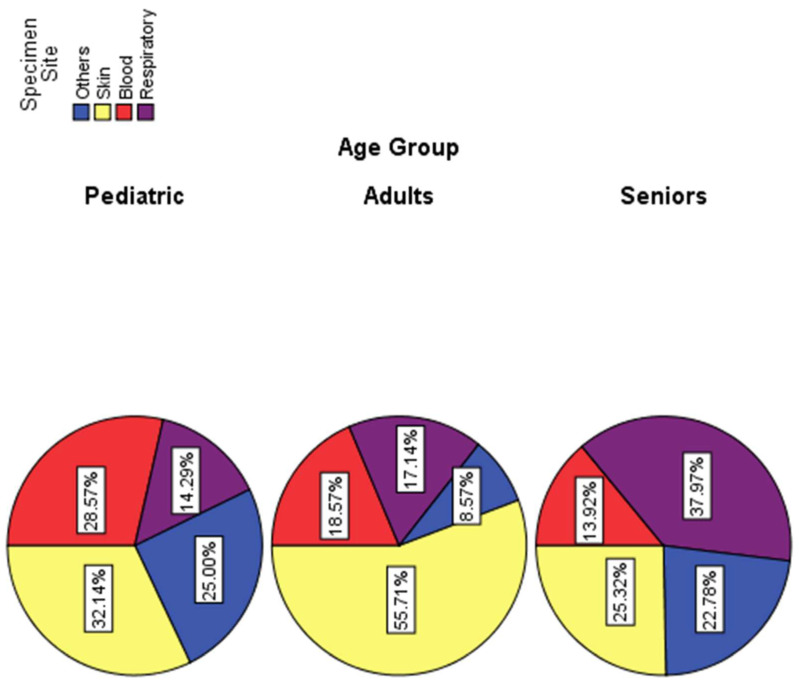
Skin and other organ-specific distribution of *Staphylococcus aureus* isolates among different age-groups of patients in Ha’il region, Saudi Arabia.

*S. aureus* is widely known to cause endogenous infections seeded from specific reservoir sites. In this study, we also intended to examine and identify this situation by studying age- and organ-specific distributions in hospitals. We also wanted to understand the potential influence of *S. aureus* carriage sites, i.e., skin and nares sites, as reservoirs for endogenous respiratory infections. For these purposes, we selected 177 patients from different age groups. As shown in Table 2, statistical analysis (Pearson Chi-Square, Likelihood Ratio Linear-by-Linear Association) revealed a significant association between age groups and infection sites. In this study, skin infections remained higher in all age groups as followed: pediatrics 32.14%, adults 56%, and seniors 25% (Figure 2). However, respiratory infections remained relatively lower in pediatrics (14.3%) and adults (17%), while seniors showed the highest infection rates (38%). Blood and “other” (organ sources other than specified) infections were higher in pediatrics (28.6% and 25%, respectively) and slightly lower in adults (18.6%) and (8.6%) and seniors (14%) and (22.8%), respectively.

### 3.3. Association between Site of Infection and S. aureus Lineage

**Figure 3:**
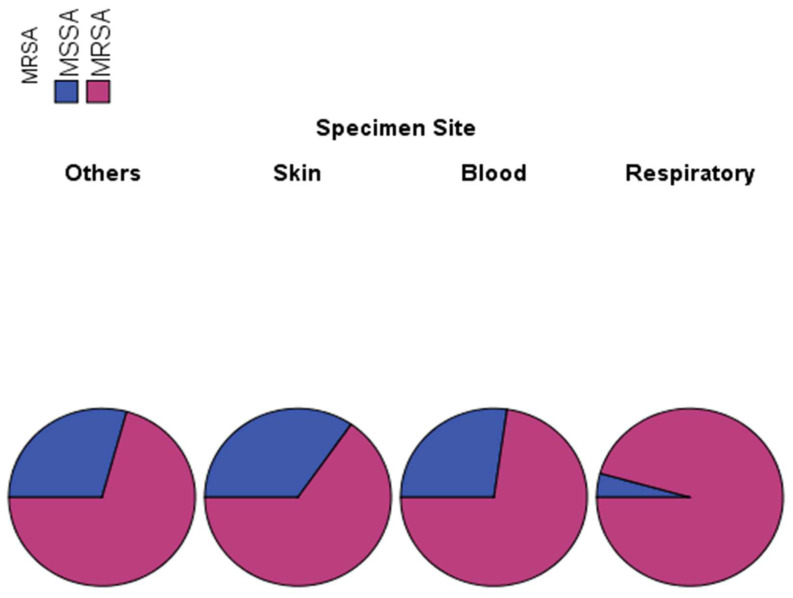
Frequencies of MRSAs and MSSAs rates on the skin and different specimen sites in Ha’il region, Saudi Arabia. Abbreviations/A- not available; MRSA—Methicillin-resistant Staphylococcus aureus; MSSA—Methicillin-sensitive Staphylococcus aureus.

To understand the clinical patterns of MRSA and MSSA lineage infections among different age groups of patients, we have developed a strategy to validate the notion of a site- and lineage-specific infection concept. For this, we have screened 195 *S. aureus* clinical isolates from different specimen types. As shown in Table 3, a significant association between specimen site and MRSA detection was observed (*p*-vlaue 0.002). Total MRSA and MSSA infections in all sites were 74.9% and 25.1%, respectively. The frequency of MRSA infections in different specimens were 70.7%, 65.3%, 72.7%, and 95.7% in “others”, skin, blood, and respiratory samples, respectively. On the other hand, MSSA infections were 29.3%, 34.7%, 27.3%, and 4.3% in “others”, skin, blood, and respiratory samples, respectively (Figure 3; Table 3).

### 3.4. Molecular Characterization and Antimicrobial Susceptibility Testing (AST) of Clinical S. aureus Lineages

Molecular characterization involving minimum laboratory processing was an innovated approach that successfully recovered specific isolates with potentially intact genomes and expression profiles reflective of the host-microenvironment during infection. This is because the samples were immediately injected into the all-in-one self-contained cartridges with built-in primers and reagents for fully robust automated GeneXpert real time-PCR that has minimum lab processing and personnel involvement. As a result, all *S. aureus* isolates were directly detected and confirmed as *S. aureus* species by the *nun* gene and differentiated MRSA lineages from MSSA by, *spa*, *mecA,* and the *mec* (SCCmec) gene sequences from specimens of patients following manufacturers recommendations. This approach ensured accurate molecular detection and minimizing the potential mutation or adaptive expressions that are prone to alter clonal properties of isolates. Concomitant culturing was carried out from all positive specimens and isolates were immediately stored at −80 °C freezer for future studies.

**Figure 4:**
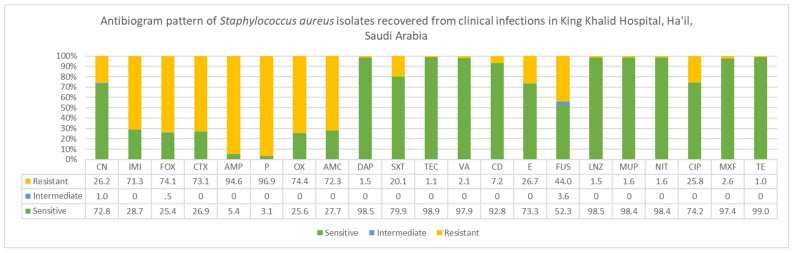
Antibiogram patterns of *Staphylococcus aureus* isolates recovered from clinical infections in Ha’il regin, Saudi Arabia. Abbreviations of 21 different antimicrobials: CN gentamicin, IMI imipenem, cefoxitin FOX, cefotaxime CTX, AMP ampicillin, *p* penicillin, OX oxacillin, AMC AMPICILLIN/SULBACTAM, DAP daptomycin, SXT trimethoprim/sulfamethoxazole, TEC teicoplanin, VA vancomycin, CD clindamycin, E erythromycin, FUS fusidic acid, LNZ linezolid, MUP mupirocin, NIT nitrofuran, CIP ciprofloxacin, MXF moxifloxacin, TE tetracycline.

The AST of clinical *S. aureus* recovered from different clinical specimens is shown Figure 4. Out of 195 *S. aureus* isolates studied, MRSA isolates were 75% (*n* = 146) while MSSA were 25% (*n* = 49). The antimicrobial susceptibility testing revealed significant progress and encouraging results for the MRSA and CA-MRSA management strategies introduced. Every patient, in-patient, out-patient, causal visitors, and consultees are all subjected to nasal screening for MRSA. Any positive result is immediately subjected to a quarantine isolation until cleared. In this study, the antibigram of *S. aureus* (Figure 4,) was tested again for 21 antimicrobials in different categories. We identified three different patterns of susceptibilities that were grouped into three different groups as followed: Group 1 antibiotics included (Tetracycline, Teicoplanin, Daptomycin, Linezolid, Mupirocin, Nitrofurantoin, Vancomycin, Moxifloxacin and Clindamycin) with a sensitivity rate of more than 97% except for Clindamycin 92%. Group 2 antibiotics included (Trimethoprim/sulfamethoxazole, Ciprofloxacin, Erythromycin, and Cefotetan/Cefalexin) with a sensitivity of more than 70%. Group 3 antibiotics included (Fusidic acid, Imipenem, Amoxicillin clavulanic acid, Cefotaxime, Oxacillin, Cefoxitin, Ampicillin and Pencillin G) with a resistance more than 50% even reaching up to 90%. Antimicrobial resistance classifications of *S. aureus* lineages are based on standard definitions for acquired resistance where they were MRSA lineage is classified as a multi drug-resistant (MDR) by virtue of only being an MRSA.

## 4. Discussion

In this study, we have investigated different aspect of nosocomial *S. aureus* infections that revealed findings with significant clinical implications for development of effective patient management strategies and MRSA and MSSA containment practices. These included the patterns and distribution of clinical isolates, rates, and frequencies of different molecular types of circulating MRSA and MSSA lineages. In addition, we studied age-specific distribution rates among groups of patients, antibiogram patterns across 21 different antimicrobials. More importantly, studies on skin carriage status for potential endogenous infections in respiratory and other sites were examined. Finally, *S. aureus* lineage-specific distributions in different organs was determined. Management of infections by different types of *S. aureus* is extremely difficult in clinical settings. This is primarily due to the elaborate mechanisms for rapid human adaptation, several modes of transmission dynamics, and the widespread pan-resistances. Therefore, present study is required for profiling of local ecotypes of circulating *S. aureus,* sources of infections, demographics, epidemiology and transmission dynamics. This study provides more insights into age and source specific distribution of dominant types of *S. aureus* in hospital settings.

Age-specific infections among different age groups of patients revealed that senior patients over 50 years old had the highest *S. aureus* infection rates (94.7%); the overwhelming of these were MRSA (81.99%) and 12.7% were caused by MSSA. This finding is consistent with many other studies in geriatric infection and long term care facilities (LTCF) around the world [50,51,52,53] However, it was surprising to find lower MRSA transmissions in hospitalized seniors compared to new admission screening and shorter-term residents. These novel findings are rare except for a few studies describing similar results in Japan [52]. At present, we do not have clear explanations on these findings other than the stricter MRSA screening protocols in place since the aftermath of MRSA pandemics more than a decade ago [6,11,12]. Positive impact of active MRSA screening protocols have been found in different countries where reduction rates were significant [54]. The recent implementation of a National Infection Control Campaign in UK resulted in large decreases in ICU-acquired infections, including MRSA, occurred across the UK ICU network during the first few years [55]. Nevertheless, state mandated State-Mandated Active Surveillance was not successful in some regions in the USA due to limitations in application [56]. However, 46.5% of the geriatric MRSA infections, and only about 4.6% MSSA, we reported here were in ICU. Unfortunately, MRSA sepsis and complications remain as a significant issue in ICU in many geographic regions where prevalence has been found exceptionally high in many countries including China [57], Korea [58], India [59], as well as Saudi Arabia [60]. Thus, further surveillance programs and stricter screening procedures have become imperative in clear MRSA from critically ill patients. This should also include non-ICU MRSA where the rates showed 35.48%.

Profiles of *S. aureus* infections in children and young adults (age groups 0 to 20 years old), showed unique distribution pattern despite their lower rates than those of older age groups. While MRSA lineage showed similar frequency in both ICU and non-ICU patients (13.9%, 9.7%, respectively), MSSA infection rates in ICU (11.6%) were similar to those of MRSA, but lower in non-ICU (4%). Since geriatric and pediatric ICUs are separate, and that MRSA screening is equally applied in all patients, the similar frequency of MRSA in ICU and non-ICU is potentially a host- organ-, and age-specific factor in colonization and/or infection rather than underlying cause for hospitalization. Relevant findings in this context have been historically established. For example, significant association between *S. aureus* types and age as well as host-and-tissue specificity has been found long ago in human and animal lineages [41,61]. This is further supported by our results in this study where Chi-Square Tests revealed significant association between age groups and infection sites (*p*-value 0.000) and that skin infections remained higher in all age groups as followed: pediatrics 32.14%, adults 56%, and seniors 25% implying carriage and genetic transfer of virulence. Intriguingly, this interesting picture also reflects reservoir and carriage status for evolutionary origins of these lineages. It is plausible that in a confined age group under a single setting the common genetic background allowed for gene transfer and evolution of virulence between MSSA and MRSA lineages. We have previously established that the host- and organ-specificity of related *S. aureus* lineages drives gene transfer and evolution of virulence [45]. This occurs only between related lineages due to a novel lineage-specific type-I restriction-modification system that blocks distant gene transfer into *S. aureus* [62]. Future vertical genomic profiling for gene content would provide valuable clues to the multifactorial mechanisms involved. This is important since MRSA rates were 2-fold higher in non-ICU (35%) than that of ICU (18.6%) among mid-aged patients (20–50 years old) while MSSA rates remained relatively lower in both settings.

The lineages specificity and infection profiles to different sites revealed further inside into the distribution of clinical isolates of the MRSA and MSSA infections. We report on significant association between specimen site and MRSA detection as indicated by the Chi-Square Tests value (0.002). The likely reason for this could be explained in terms of the natural resident sites on human skin and respiratory regions (anterior nares and nasopharyngeal sites) and their transmission routes therefrom. Consequently, MSSA infections rates were higher on skin implying potential carriage owing to the fact that skin is a normal ecological niche. Since MRSA detection in respiratory sites including all nasopharyngeal and lung regions as well as in blood is common, its high frequency on skin among all age groups might imply endogenous seeding from reservoir carriage sites. This particularly strengthened the contagious cutaneous mode of transmission route in nosocomial MRSA dynamics. Future molecular genotyping and genomic analysis will provide more insights into MRSA evolutionary origins and MSSA profiles on carriage sites.

The patterns of *S. aureus* antibiogram shown by the antimicrobial susceptibility testing (AST) indicated effectiveness of the MRSA management strategies introduced. All isolates were tested again 21 antimicrobials in different categories. By test results as being considered MRSA, isolates were assigned a MDR definition based on standard classifications of MRSA [48]. We identified three different patterns of susceptibilities that were grouped into three different groups as followed: Group 1 antibiotics included (Tetracycline, Teicoplanin, Daptomycin, Linezolid, Mupirocin, Nitrofurantoin, Vancomycin, Moxifloxacin and Clindamycin) with a sensitivity rate of more than 97% except for Clindamycin 92%. Group 2 antibiotics included (Trimethoprim/sulfamethoxazole, Ciprofloxacin, Erythromycin, and Cefotetan/Cefalexin) with a sensitivity of more than 70%. Group3 antibiotic included (Fusidic acid, Imipenem, Amoxicillin clavulanic acid, Cefotaxime, Oxacillin, Cefoxitin, Ampicillin and Pencillin G) with a resistance more than 50% even reaching up to 90%. Susceptibility to Group 1 is much higher prompting to CA-MRSA phenotypes. As a widely accepted property, these lineages are normally susceptible to non beta lactam antibiotics. Future studies on genotyping and detection of gene content and cassette types would reveal more information.

## 5. Conclusions

In this study, we provided significant insight into the distributions of *S. aureus* among different age groups, specimen types and sites of infection, hospital units, disease patterns, and associated lineage types. The highest prevalence of MRSA in senior patients at ICU is worrisome. However, the intriguingly similar coexistence of MRSA and MSSA in younger groups < 20 years at ICU, and the subsequent two-fold increase in MRSA at non-ICU patients over 20 years strongly imply age-specific selection for the evolution of resistant strains from resident MSSA ancestors. More importantly, the highly significant association to infection sites, particularly, the dominance of skin infections has a high potential for skin-carriage. These findings have important clinical implications for strategic planning in patient management and *S. aureus* control, particularly in geriatric and pediatric settings. Future vertical studies will reveal more insights into lineage/types, gene content, and evolutionary patterns. This study is limited by being single-centered; a large scale multi-center approach is likely to gain more insights into *S. aureus* infection profiles in the whole region.

## Figures and Tables

**Table 1:** Profiles of nosocomial MRSA and MSSA clinical isolates among different age-groups of patients at ICUs, and other non-ICUs settings in Ha’il region, Saudi Arabia.

*Staphylococcus aureus* Isolates			Age Groups		
			<20	20–50	>50
MSSA	Setting	ICU	5	2	9
		Non-ICU	5	10	25
	Total		10	12	34
MRSA	Setting	ICU	6	8	34
		Non-ICU	12	43	99
	Total		18	51	133
Total	Setting	ICU	11	10	43
		Non-ICU	17	53	124
	Total		28	63	167

Abbreviations/A- not available; MRSA—Methicillin-resistant *Staphylococcus aureus*; MSSA– Methicillin-sensitive *Staphylococcus aureus.*

**Table 2:** Frequency of *Staphylococcus aureus* isolate from skin and other organs among different age-groups of patients in Ha’il region, Saudi Arabia.

			SPECIMEN SITE				Total
			Others	Skin	Blood	Respiratory	
Age	<20	Count	7	9	8	4	28
		Percentage	25.0%	32.1%	28.6%	14.3%	100.0%
	20–50	Count	6	39	13	12	70
		Percentage	8.6%	55.7%	18.6%	17.1%	100.0%
	>50	Count	18	20	11	30	79
		Percentage	22.8%	25.3%	13.9%	38.0%	100.0%
Total		Count	31	68	32	46	177
		Percentage	17.5%	38.4%	18.1%	26.0%	100.0%
	Value	df	Asymptotic Significance (2-sided)				
Pearson Chi-Square	25.032	6	0.000				
Likelihood Ratio	25.237	6	0.000				
Linear-by-Linear Association	2.848	1	0.051				
N of Valid Cases	177						

**Table 3:** Association rates between site of infection on different specimen and MRSA and MSSA lineages in Ha’il region, Saudi Arabia.

Specimen Site by			*Staphylococcus aureus* Isolates		Total
			MSSA	MRSA	
Specimen Site	Others	Count	12	29	41
		Percentage	29.3%	70.7%	100.0%
	Skin	Count	26	49	75
		Percentage	34.7%	65.3%	100.0%
	Blood	Count	9	24	33
		Percentage	27.3%	72.7%	100.0%
	Respiratory	Count	2	44	46
		Percentage	4.3%	95.7%	100.0%
Total		Count	49	146	195
		Percentage	25.1%	74.9%	100.0%

## Data Availability

There is no additional data deposited on any other site other than those in this manuscript.
